# Tooth Extraction and Its Possible Relation to the Pregnant State

**Published:** 1879-01-20

**Authors:** Garrett Newkirk

**Affiliations:** Wenona, Ill.


### *TOOTH-EXTRACTION AND ITS POSSIBLE RELATIONS TO THE PREGNANT STATE.*

BY GARRETT NEWKIRK, M. D. WENONA, ILL.

Among the "Hints and Queries" in the *Dental Cosmos* questions sometimes appear in such form as seemingly to show an utter incapacity of the querist to comprehend the extent and scope of a proper answer. Solutions to many-sided and far-reaching problems are sought for in a simple yes or no.

In medicine, there has been a reaching out after *specifics*, something which should enable the physician to deal with intricate and ever-varying problems of life and death, not by inductive reasoning, but by the mathematical routine prescription of drugs. So, in dentistry, some are seeking exact specific rules of action for cases involving a great variety of considerations, by which rules they seek are made impossible.

Where circumstances alter cases so generally that no two can be precisely similar, where each requires a special study of itself in order to a correct understanding, too much caution can hardly be exercised in creating arbitrary rules.

In the February (1878) number of the *Dental Cosmos* "J. B. W." asked with reference to the facts as to the extraction of teeth for pregnant women. The only answer given was by some one who said that in a practice of over thirty years he had never hesitated to extract teeth for women in all stages of pregnancy, and, so far as he knew without any unfavorable results; the inference from his answer being, that in considering the question of the extraction of any woman's teeth, it makes no difference whether she be pregnant or not.

From this conclusion I most respectfully dissent. Were a dentist considering that question with reference to a friend of mine, I should prefer that he would not take that view of it and act accordingly. I believe there *is* a difference—an important one—that ought to be taken into account, and which should exercise a conservative influence on our decision in these cases; how far will appear as we proceed.

In considering any operation or treatment proposed for the pregnant woman, there should be constantly borne in mind the intimate relationship which exists between the mother and the child, and the possible influence that may be exercised upon that relationship by the course proposed. We must remember that whatever affects the mother, whether of a physical or mental character, may also affect the child, either favorably or otherwise.

The question for us to consider in this connection is. What may be reasonably expected as a probable or possible result of tooth-extraction during pregnancy? Is this condition to be practically ignored? May it be in any instance the possible cause of a miscarriage or other injury?

The causes of miscarriage may be any kind of violence, direct or indirect, accidental or intentional; idiosyncrasy; disease of a general or local character, and reflex influences of an injurious nature, whether mental, moral, or purely nervous,—whether acting directly through uterine contractions, or indirectly through the circulation upon the fœtus.

It is with the last class that we are here principally concerned. It may be remarked in the first place that many abortions cannot be positively accounted for. An abortive habit (predisposition) exists in some cases, requiring only a slight cause to make it active. A woman may be the subject of an apparently slight disturbing cause, avoidable or otherwise, a misstep, a blow, an extra muscular effort, a fright, a shock, a mere surprise, an unusual emotion, a sudden pain, and soon thereafter a miscarriage follows. In a large majority of these casese what the immediate exciting causes are can only be conjectured. All that we can say is that the mis-step, fall, surprise, shock, etc., ought to have been avoided. It must also be borne in mind that effects may be, and often are, produced that *are important in their character and yet come short of producing abortion.* There are children born that have been *almost aborted*, and many more that have been subjected to causes having an unfavorable influence upon their harmonious and healthy development.

There are certain nervous temperaments, certain idiosyncrasies and special habits, with reference to which, in the pregnant woman, it cannot be said with certainty that any considerable disturbing influence superadded will not produce bad results. An abortion may follow, or short of that, injury of some sort to the child. It is well, therefore, not to trifle under such circumstances, or we may some day be guilty of having produced mischievous results; certainly we take a risk, even though it be but one in a hundred,—one which we ought not to take except the circumstances absolutely demand it.

Now, what does tooth-extraction necessarily and possibly involve? Physically, it involves a solution of continuity of from one to three square inches of surface of living tissue; the sudden rupture of a large number of small blood-vessels, and from one to four nerves. Contingently, it may involve a fracture more or less extensive of bony process, unusual suffering, or loss of blood. It produces invariably upon the conscious subject a sudden nervous impression,—*shock*,—varying from trifling to serious. Pain, for a moment almost unendurable; semi-involuntary, possibly violent movements and outcries, followed by faintness and nervous tremor, are the coincidents and sequelæ in some degree in a majority of cases. Additionally, there may be fear, fright, and occasionally, though rarely, uncontrollable anger.

The degree of shock likely to ensue may be in a measure anticipated by attention to the following considerations, viz.: the temperament; present state of health, especially as it pertains to the nervous system; the character, history, and present condition of the tooth in question; the state of the neighboring tissues, and the probable ease or difficulty of extraction. The mental condition of patient should also be considered.

The order which temperaments bear to shock is about as follows: 1, the nervous; 2, nervo-sanguineous; 3, nervo-bilious; 4, nervo-lymphatic; 5, bilious; 6, lymphatic.

The condition of the nervous system at the time of an operation has a marked influence on results; that which may be well borne at one time, may at another be attended by a severe shock, and followed by serious prostration. This fact is never more apparent than in the extraction of teeth. A want of sleep, severe pain (especially if paroxysmal), unusual emotion or excitement, severe illness or overwork, may produce a state of exalted nervous sensibility, or rather irritability, highly unfavorable for an operation.

As to the tooth itself, Has it an inflamed pulp? Is it dead? Is there alveolar trouble of any kind? Is it particularly sensitive to instrumental touch? Has it one, two, three, or four roots and nerves? Is the trouble of reflex, malarial, or neuralgic origin? Would much force be required in its extraction? Is it probably amenable to therapeutic treatment? These are some of the questions which may be asked with reference to such cases where extraction is proposed, and which ought to be correctly determined by the dentist before he assumes the responsibility of performing the operation. It is not to be looked upon as a trifling operation simply because it is so common. These questions, always proper, become of greater importance whenever the case in hand is that of a pregnant female.

I believe the following rules to be in the line of ordinary prudence. Where a choice has to be made between allowing the tooth to remain, involving odontalgia, severe neuralgia, antral or alveolar abscess,—

conditions compromising the general health and comfort of the patient, and the removal of the offender, the latter course is the proper one; but this is a contingency not often arising, and may usually be avoided.

If the operation seems to be inevitable, however, and the temperament, mental and other conditions are unfavorable, with undue nervous irritability, it would be better to modify these conditions, either by forced rest and sleep under an opiate previous to, or by partial anææsthesia at the time of extracting.

During the pregnant state no tooth should be extracted to please the patient or her friends, or to prepare the mouth for artificial work. It should be only as a choice of two well recognized evils, and then especial care should be exercised to avoid nervous impressions as far as possible.

As the greater danger of miscarriage exists during the earlier months of pregnancy, more particularly the third and fourth, temporary treatment, protection or filling, should, if possible, be resorted to if only for a few weeks.

Again, we all know that with certain timid people the sight of an operating-chair, of instruments, the touch of cold steel, or the sight of blood may, any of them, be sufficient to cause an almost insupportable condition of nervousness and dread. This is particularly true of females, and especially when *enciente*. Hence the increased necessity for trying to avoid or to modify these disturbing causes. Under some circumstances it would be better for the dentist to visit the patient at her house, and extract the tooth with an instrument previously warmed and kept from her sight. Much nervous distress may in this way be avoided.

I have been thus careful to call attention to these well-known facts, because explicitness seemed to be demanded by the character of the question.

I do not think that any practitioner of dentistry should entertain the theory that the pregnant state is one that may safely be ignored, for I believe such a theory is full of possible dangers. Nor should it be forgotten that the question of miscarriage is not the only one involved in this matter. *Prenatal influences* are recognized by intelligent observers, both in and out of medical circles, as among the most important in determining the organic qualities of human beings, and more than usual care is exercised by sensible people to avoid disturbing influences upon the woman with child.

Should the dentist, who ought to be a physiologist, be less regardful of safe rules than those who have been taught only by observation and the exercise of ordinary common sense?

It is hoped that these reflections may not be without value, especially to the younger members of the profession, whose attention may not have been called particularly to the subject.—*Cosmos.*

